# Large-scale neuroanatomy using LASSO: Loop-based Automated Serial Sectioning Operation

**DOI:** 10.1371/journal.pone.0206172

**Published:** 2018-10-23

**Authors:** Timothy J. Lee, Aditi Kumar, Aishwarya H. Balwani, Derrick Brittain, Sam Kinn, Craig A. Tovey, Eva L. Dyer, Nuno M. da Costa, R. Clay Reid, Craig R. Forest, Daniel J. Bumbarger

**Affiliations:** 1 Georgia Institute of Technology, G. W. Woodruff School of Mechanical Engineering, Atlanta, GA, United States of America; 2 Georgia Institute of Technology, School of Electrical and Computer Engineering, Atlanta, GA, United States of America; 3 Allen Institute for Brain Science, Seattle, WA, United States of America; 4 Georgia Institute of Technology, H. Milton Stewart School of Industrial & Systems Engineering, Atlanta, GA, United States of America; 5 Georgia Institute of Technology, Coulter Department of Biomedical Engineering, Atlanta, GA, United States of America; University of Campinas, BRAZIL

## Abstract

Serial section transmission electron microscopy (ssTEM) is the most promising tool for investigating the three-dimensional anatomy of the brain with nanometer resolution. Yet as the field progresses to larger volumes of brain tissue, new methods for high-yield, low-cost, and high-throughput serial sectioning are required. Here, we introduce LASSO (Loop-based Automated Serial Sectioning Operation), in which serial sections are processed in “batches.” Batches are quantized groups of individual sections that, in LASSO, are cut with a diamond knife, picked up from an attached waterboat, and placed onto microfabricated TEM substrates using rapid, accurate, and repeatable robotic tools. Additionally, we introduce mathematical models for ssTEM with respect to yield, throughput, and cost to access ssTEM scalability. To validate the method experimentally, we processed 729 serial sections of human brain tissue (~40 nm x 1 mm x 1 mm). Section yield was 727/729 (99.7%). Sections were placed accurately and repeatably (x-direction: -20 ± 110 μm (1 s.d.), y-direction: 60 ± 150 μm (1 s.d.)) with a mean cycle time of 43 s ± 12 s (1 s.d.). High-magnification (2.5 nm/px) TEM imaging was conducted to measure the image quality. We report no significant distortion, information loss, or substrate-derived artifacts in the TEM images. Quantitatively, the edge spread function across vesicle edges and image contrast were comparable, suggesting that LASSO does not negatively affect image quality. In total, LASSO compares favorably with traditional serial sectioning methods with respect to throughput, yield, and cost for large-scale experiments, and represents a flexible, scalable, and accessible technology platform to enable the next generation of neuroanatomical studies.

## Introduction

**Fig 1:**
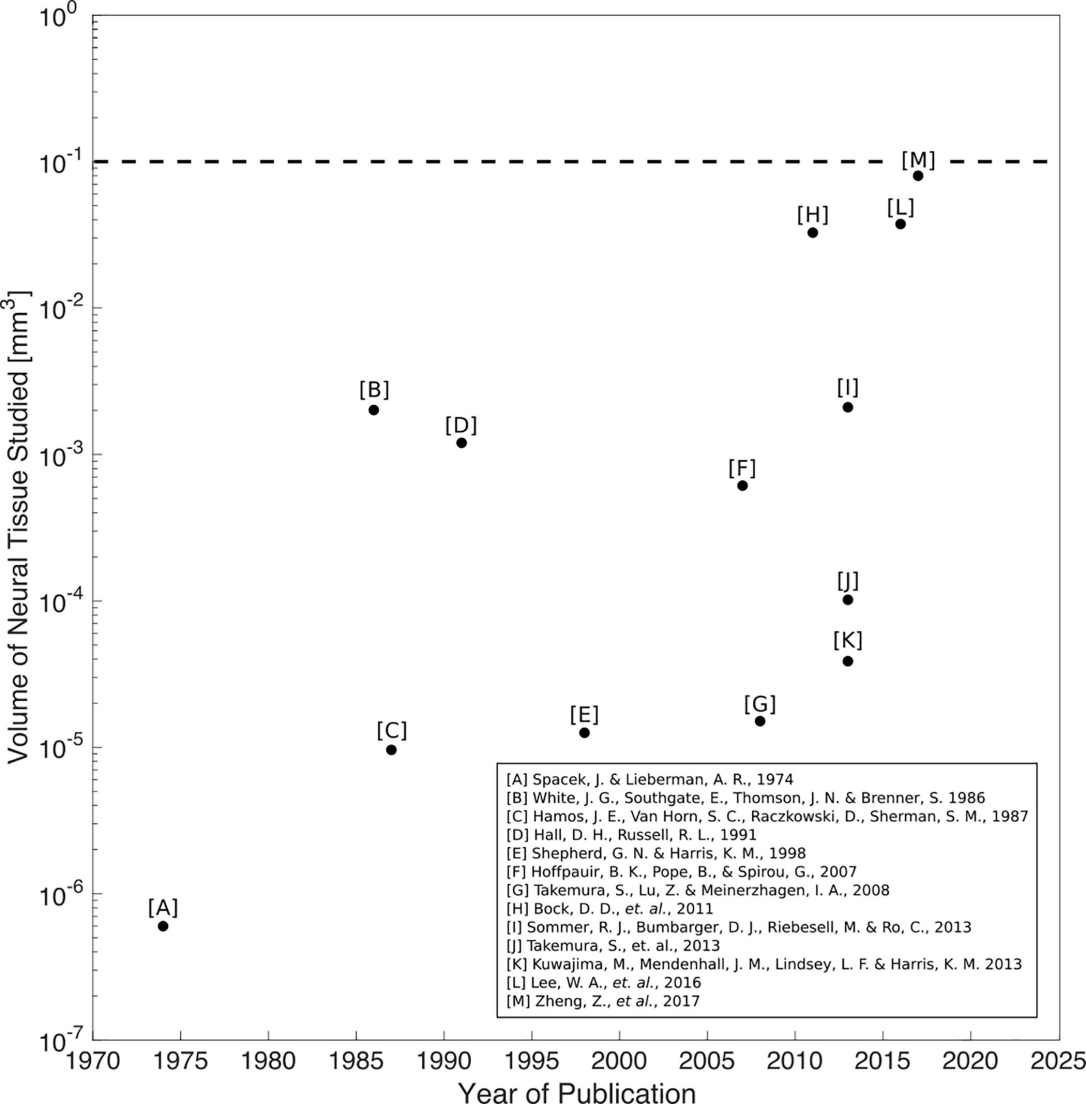
Volume of neural tissue in ssTEM studies versus publication year. Each data point represents one journal publication that used ssTEM for neuroanatomical studies. We observe the general trend of increasing neural tissue volume studied over time. The largest neuroanatomical ssTEM study (reference M Zheng, Z., *et*. *al*., 207) to date remains an order of magnitude below a cubic millimeter.

Serial section transmission electron microscopy (ssTEM) is the most promising tool for investigating the three-dimensional structure of the brain with nanometer-scale resolution [1–3]. In recent years, ssTEM studies have provided significant insight into the physiology and neuroanatomy of mammalian and non-mammalian nervous systems with resolution and scope previously not possible [4–6], (respectively, references I, L, M in Fig 1). From published ssTEM literature, we observe a general trend of increasing neural tissue volume studied over time, exemplifying the scientific interest in the field to study larger and larger volumes of neural tissue, as shown in Fig 1. Yet, a significant challenge remains in the *scalability* of ssTEM. As the volume of brain tissue to be studied grows larger, does ssTEM remain a viable technology in terms of yield, cost, and throughput?

Currently, the ssTEM workflow is composed of five primary steps: (1) bulk tissue processing, (2) serial sectioning, (3) post-staining, (4) TEM imaging, and (5) image segmentation. In recent years, significant advances in bulk tissue processing, post-staining, TEM imaging, and image segmentation have shown the potential scalability of ssTEM [5, 7–13]. In contrast, traditional techniques and methods for serial sectioning for ssTEM have remained unchanged for nearly 60 years [14, 15]. A recent advancement in serial sectioning technology, called the Automatic Tape-collecting Lathe Ultramicrotome (ATLUM), shows promise for the automation of serial sectioning, but demonstration and characterization for neuroanatomical ssTEM studies remains to be shown [16, 17]. Accordingly, advances in methods for serial sectioning will be necessary for the scalability of ssTEM.

**Fig 2:**
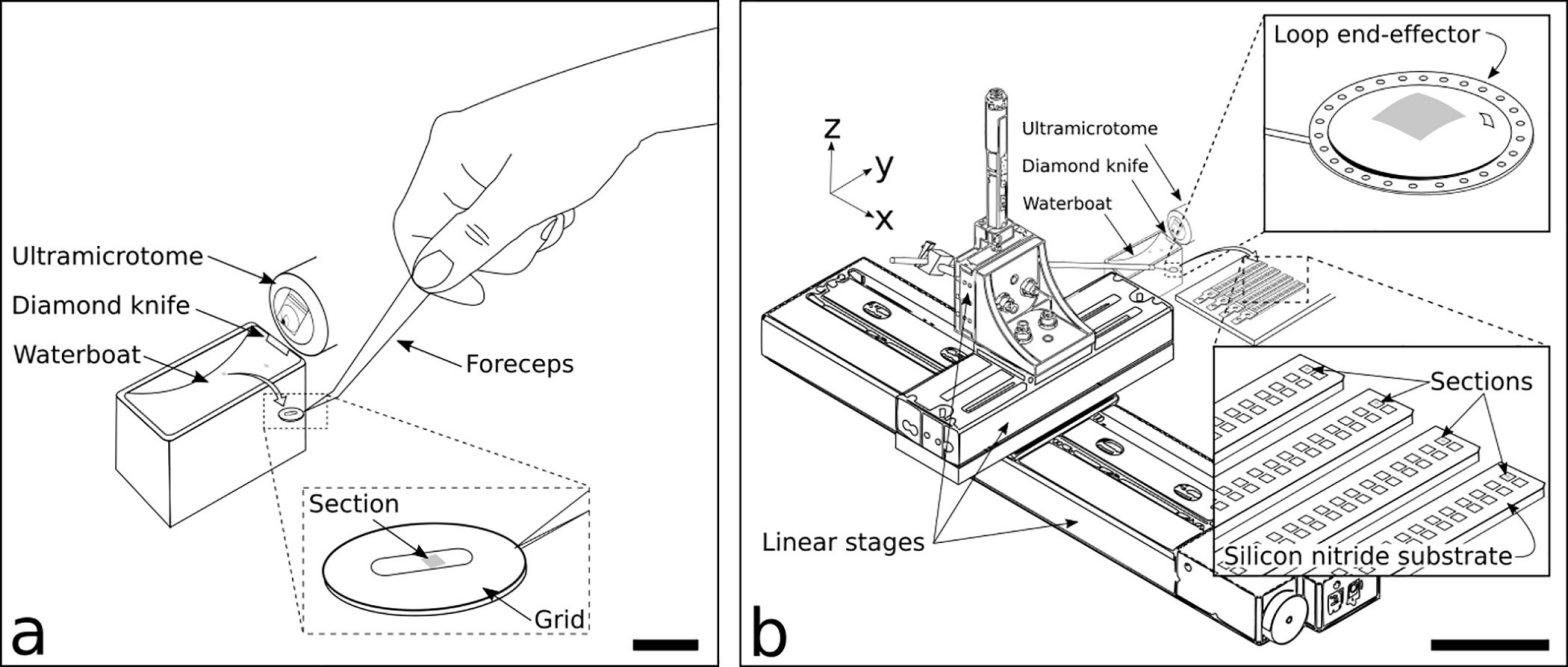
Traditional serial sectioning (*left*) as compared to LASSO (*right*). **(a)**. Sections are cut on an ultramicrotome using a diamond knife and slide into an adjoining water-filled waterboat, where they float on the water surface. Using a TEM grid held by forceps, a skilled user picks up section(s) from the waterboat onto a TEM substrate, e.g., grid (*Fig 2A*, *inset*). Scale bar: 10 mm. **(b)** For LASSO, sections are fabricated in an identical manner as in Fig 2A, using the same ultramicrotome, diamond knife, and waterboat. From the waterboat, sections are picked up using a loop end-effector, actuated via a robotic system composed of three orthogonal linear axes. Held in the loop end-effector by surface tensions forces (*Fig 2B*, *inset*, *top right*), the section is placed onto microfabricated silicon nitride substrates (*Fig 2B*, *inset*, *bottom right*). Multiple sections are placed onto the same substrate, with each section having its own imaging aperture; a set of substrates compose a “batch,” (e.g., 4 substrates = 1 batch, as shown in Fig 2B, inset, bottom right). Scale bar: 100 mm.

In traditional serial sectioning, an ultrathin (< 40 nm) brain slice (or “section”) is cut with a diamond knife into an attached water reservoir, i.e., “waterboat” (see Fig 2A). Individual sections are typically ultrathin in order to have sufficient out-of-plane resolution during EM tomography. Once cut, the section is suspended on and then transported (e.g., by manually whisking the water’s surface with an eyelash) on the water’s surface to a suitable pickup location [15]. Subsequently, the section is carefully picked up by hand (e.g., by using a loop end-effector; see [18–20] for previous loop-based serial section pick-up methods) and placed onto a TEM substrate (e.g., copper, plastic-coated slot-grid), paying close attention to align the section with the substrate aperture (see [15] for further details on traditional serial sectioning methodology.) Thus, for traditional serial sectioning, the manual pickup of sections must be repeated for each section without error. From prior work, experienced investigators typically experience 1–3% section loss; with regards to cost, each TEM grid costs ~40 cents; with regards to throughput; each cycle of ultrathin sectioning—transporting, picking up, and placing the section—takes approximately 2 min [4, 5, 13, 15]. While these metrics on a section-by-section basis may seem reasonable, it is the multiplication (or “scaling”) of these values by tens of thousands of serial sections that prohibits large ssTEM neuroanatomical studies.

In the field of industrial engineering, batch processing, i.e., the production of goods in quantized groupings (or “batches”), is a common methodology for yield, cost, and throughput optimization for scaling manufacturing processes [21–24]. First introduced by the Toyota Motor Company in the late 1930’s to compete with continuous processing methods (e.g., the assembly line), batch processing enables high-yield, low-cost, high-throughput production [24]. We set out to explore whether batch processing may be an effective methodology for scaling serial sectioning for large neuroanatomical ssTEM studies.

We introduce conceptually, experimentally, and mathematically an alternative method for large-scale serial sectioning, termed Loop-based, Automated Serial Sectioning Operation (LASSO). In our methodology, individual sections are picked up from the waterboat and placed onto TEM substrates using robotic tools for accurate and repeatable, rapid serial sectioning. Batches of sections are placed onto custom microfabricated substrates, reducing overall handling and imaging time of sections. In total, we present a flexible, scalable, and accessible technology platform for serial sectioning to enable the next generation of large-scale neuroanatomical ssTEM studies.

## Materials and methods

In LASSO, sections are cut on a conventional ultramicrotome with a diamond knife into an adjoining waterboat, as depicted in Fig 2B. From the waterboat, sections are picked up using a loop end-effector, controlled by three orthogonal linear actuators. Held in place by water surface tension forces within the loop (Fig 2B, inset, top right), the sections are placed onto a microfabricated silicon/silicon nitride TEM substrate (Fig 2B, inset, bottom right). Each substrate holds more than one section with each section having its own imaging aperture. A set of substrates composes a “batch,” (e.g., 4 substrates = 1 batch, as shown in Fig 2B, inset, bottom right). Consecutive sections are never placed on the same batch; therefore, the probability of consecutive section loss is minimized.

### Experiment methods

**Fig 3:**
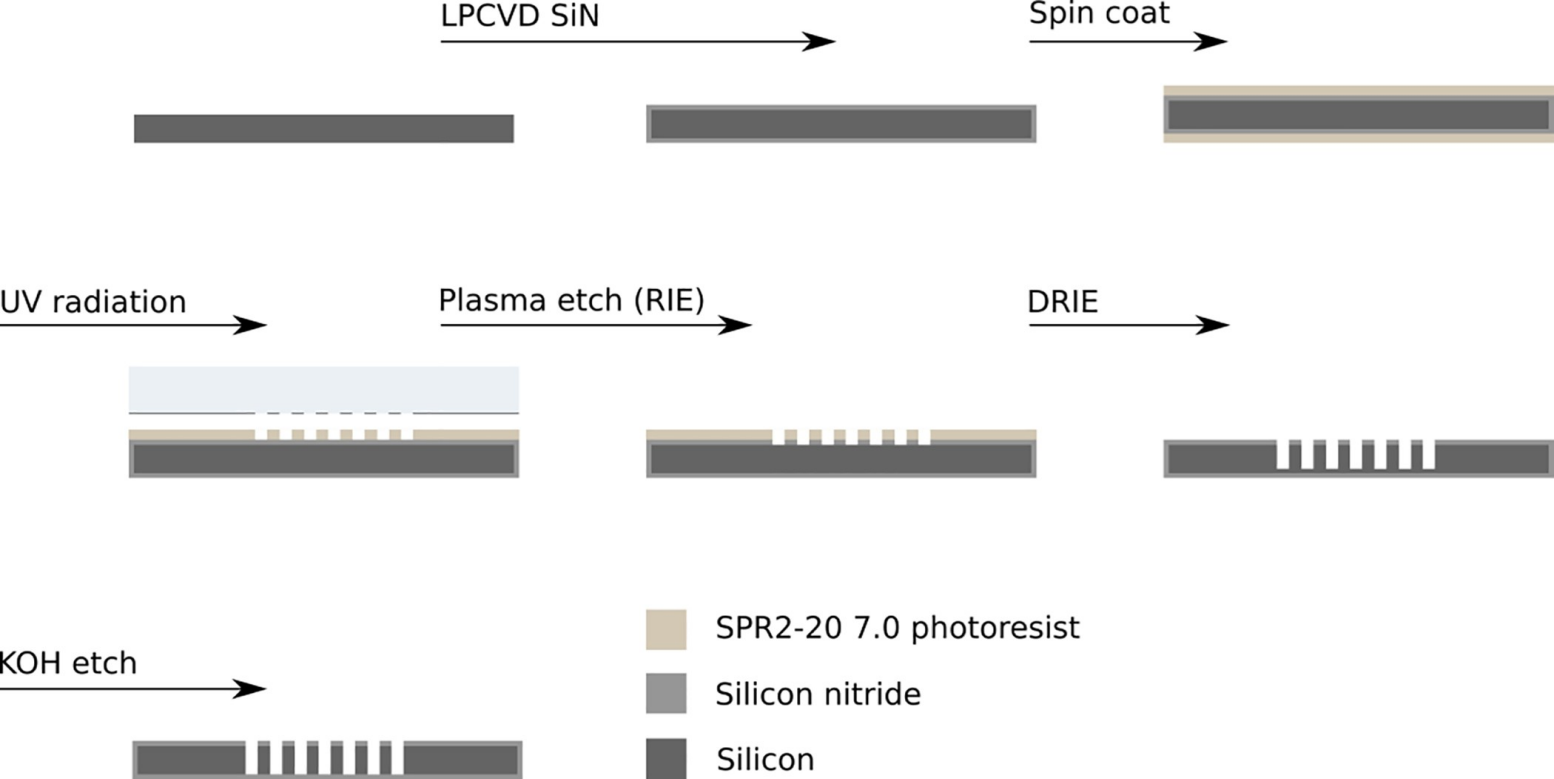
Overview of fabrication of silicon/silicon-nitride (Si/SiN) TEM substrates. Wafers are initially deposited with low-stress silicon nitride (100 nm-thick) followed by photolithography, and plasma etching. In practice we fabricated eight substrates on one 100-mm diameter wafer. See Supplemental Information for fabrication plan details.

Experimentally, microfabricated substrates were manufactured using conventional semiconductor processing techniques, outlined below in Fig 3. A detailed fabrication protocol is provided in the Supporting Information, see S1 Protocol. A single tissue block was manually trimmed to a cross sectional of ~1 mm x ~1 mm and placed into an ultramicrotome (Leica UC7). Four microfabricated substrates were placed on a hotplate (VWR), adjacent to the ultramicrotome, comprising 160 imaging apertures. The hotplate was set to 95 ºC to enable rapid (< 30 s) drying of sections once placed onto the substrates. The ultramicrotome was set at cutting speed of 0.1 mm/s. Sections were transported away from the knife-edge via a puff of air, delivered manually through an air needle placed adjacent to the waterboat.

A 3-axis robotic system (Zaber Technologies), positioned adjacent to the ultramicrotome, was used to transport sections between the diamond knife water boat (Diatome) and a microfabricated substrate, using a platinum-iridium wire loop (3 mm diameter) end-effector (TedPella) rigidly affixed to the terminal axis. Section pickup was conducted using manual control of the actuator system via an Xbox controller while placement of the sections onto the microfabricated substrates was automated, following a pre-specified array pattern matching that of the microfabricated substrate apertures. Custom Python-based software was used to interface with the Xbox controller and the actuator system.

After serial sectioning, sections were picked up onto the substrates using the loop end-effector and 3-axis robotic system, with no consecutive sections being placed on the same substrate. A movie of the section pick up and placement process is provided in the Supporting Information, see S1 Movie. Between each section, the ultramicrotome was manually paused to allow for section pickup and placement; once pickup and placement were complete, the ultramicrotome sectioning was resumed. Section pickup and placement was conducted until all 160 apertures were occupied with sections or until a 2-hour imposed time limit to prevent effects due to water evaporation.

Video recording of serial section pickup and placement was conducted to analyze processing throughput and section placement accuracy, repeatability, and yield. From these videos, a MATLAB-script was used to automatically identify and compute each section centroid. Sections that had separated into two or more pieces (but were successfully placed onto the substrate) were excluded from our accuracy and repeatability analysis to maintain a consistent section centroid definition. Yet, these partial sections were picked up and placed successfully. In determining yield, we defined section loss as sections lost due to broken substrate apertures, due to failed section pickup (e.g., loop end-effector damages the section during pickup, or partial sections were unrecoverable), due to failed section transport (e.g., surface tension within loop end-effector breaks during actuator movement), or due to failed placement (i.e., section is not released from the loop end-effector). Thus we define single section loss rate, *p*, as the probability of damaging or losing one section due to any of these occurrences. We note, in our yield calculation, we include sections that were partially misplaced over the TEM substrate imaging aperture but were successfully placed onto the substrate.

Substrates were imaged using a JEOL EX-II TEM with a camera array system (see [13] for more information regarding the camera array system). An alternative, commercially available loadlock (Voxa) was used to fixture and position the microfabricated substrates during imaging (see [25] for further details regarding the custom loadlock.)

To compare the image quality of the microfabricated films (SiN) used in LASSO with conventional TEM grids (Luxel), we used two standard metrics to quantify the detectability of neuroanatomical structures (e.g., synaptic vesicles). First, the edge spread function (ESF) was computed by manually annotating synaptic vesicles in ITK-Snap on four separate images (2 SiN, 2 Luxel), drawing a line from the vesicle exterior to the interior, and then measuring the change in pixel intensities across the vesicle boundaries [26]. Using derivative-based change-point detection, the change-points in the ESF were obtained and a line was fit to all points between the identified change points. Thus, the slope of this line, i.e., the mean roll-off of the ESF, provides a quantitative measure of the sharpness of edges for neuroanatomical structures of interest in our images [27]. To quantify image contrast, the Michelson contrast was calculated for vesicle interior versus vesicle exterior points. Again, the vesicle interior and exterior pixel intensities were obtained from manually annotated vesicles (ITK-Snap). Then, the mean contrast value across all annotated vesicles was computed, giving a quantitative metric of the detectability of neuroanatomical structures in our images.

## Mathematical modeling

### Yield modeling

As a statistical model for predicting yield, we let each section pickup and placement event be a binomial random variable, where *n* is the number of sections to be processed and *p* be the probability of failure, i.e., damaging or losing one section. To successfully reconstruct a cubic millimeter of neural tissue with 40 nm-thick sections, 25,000 consecutive sections must be cut and imaged with zero *consecutive* section loss. Sections must be 40 nm thick or less to resolve distal neuronal processes that often are ~100 nm thick, thereby spatially sampling above the Nyquist frequency [3]. From prior literature, we can expect a single-section loss rate lower bound of 1% [4, 5, 13]. The probability of losing two consecutive sections then, assuming P(1 lost section) = *p* = .01, is

P(2consecutivelost)=p*p=.0001.

Assuming n = 25,000 and a binomial probability distribution, the probably of losing 1 or more pairs of consecutive sections is

$$P(1\;or\;more\;consecutive\;pairs\;lost)=\sum_{i=1}^{n-1}(\begin{array}{c} n-1\\ i \end{array})\;{\left(p*p\right)}^{i}\;{\left(1-p*p\right)}^{n-1-i}\approx 92\%.$$

The yield, or probability of success assuming failure criteria described above, for an experiment with 25,000 sections traditionally processed is thus 1–0.92 = 8%. Thus, with traditional serial sectioning, large-scale neuroanatomical studies are expected to be impractical. For LASSO, a “batch” of sections will comprise c*m sections, where *m* is the number of substrates per batch and *c* is the number of sections per substrate. In total, there are *k* batches where

$$k=\frac{n}{c*m}$$ (1)

and *n* is the total number of section to be processed. Let us assume the loss rate of a single substrate is *p’*. This is likely an overestimate of the substrate loss rate, since as substrates become larger and easier to handle, we expect fewer substrates to be lost. Within one batch, we will mandate that no two consecutive sections be placed on the same substrate. Therefore, we must lose two or more substrates to lose two consecutive sections. This probability, *P*, can be calculated as

$$P=P(losing\;2\;or\;more\;substrates\;in\;1\;batch)={1-[(1-p^{\prime })}^{m}+\left(\begin{array}{c} m\\ 1 \end{array}\right)\;(p^{\prime })\;{\left(1-p^{\prime }\right)}^{m-1}].$$ (2)

This represents the probability of one failed batch. Furthermore, we can calculate the probability of one or more failed batches out of the total number of batches, *k*, as

$$P(1\;or\;more\;failed\;batches)=\sum_{j=1}^{k}(\begin{array}{c} k\\ j \end{array})\;{\left(P\right)}^{i}\;{\left(1-P\right)}^{k-j}.$$ (3)

Thus, Eqs 1–3 compose a framework by which to optimize yield by modulating *m*, the number of substrates per batch, and *c*, the number of sections per substrate.

### Throughput modeling

The total time to collect neuroanatomical datasets can be analyzed on a section-by-section basis. For traditional serial sectioning, the total time for data collection can be written as

$$T_{traditional}=(t_{imaging}+t_{pickup,\;traditional}+t_{load\;time,traditional})*n,$$ (4)

where *t*_imaging_ is the time to image one section, *t*_pickup, manual_ is the time to manually pick up and place one section onto a grid, *t*_load time, traditional_ is the time to load one grid into the TEM, and *n* is the total number of sections to be processed.

For LASSO, the total time for data collection can be written as

$$T_{LASSO}=\left(\frac{t_{load\;time,\;LASSO}}{c*m}+t_{imaging}+t_{pickup,\;robotic}\right)*n$$ (5)

where *t*_pickup, robotic_ is the time to robotically pick up and place one section onto a substrate, *t*_load time, LASSO_ is the time to load one substrate into the TEM.

### Cost modeling

For large sections, each TEM grid typically holds one section. As a result, the total cost for large-scale neuroanatomical datasets can be written as

$$C_{traditional}=n*c_{grid}$$ (6)

where *c*_grid_ is the cost of one grid. For LASSO, sections are placed onto microfabricated substrates, which are manufactured on a wafer-by-wafer basis.

$$C_{LASSO}=\frac{n}{s}*w$$ (7)

where *s* is the number of sections per wafer, and *w* is the cost of processing one wafer.

## Results and discussion

### Modeling results

**Fig 4:**
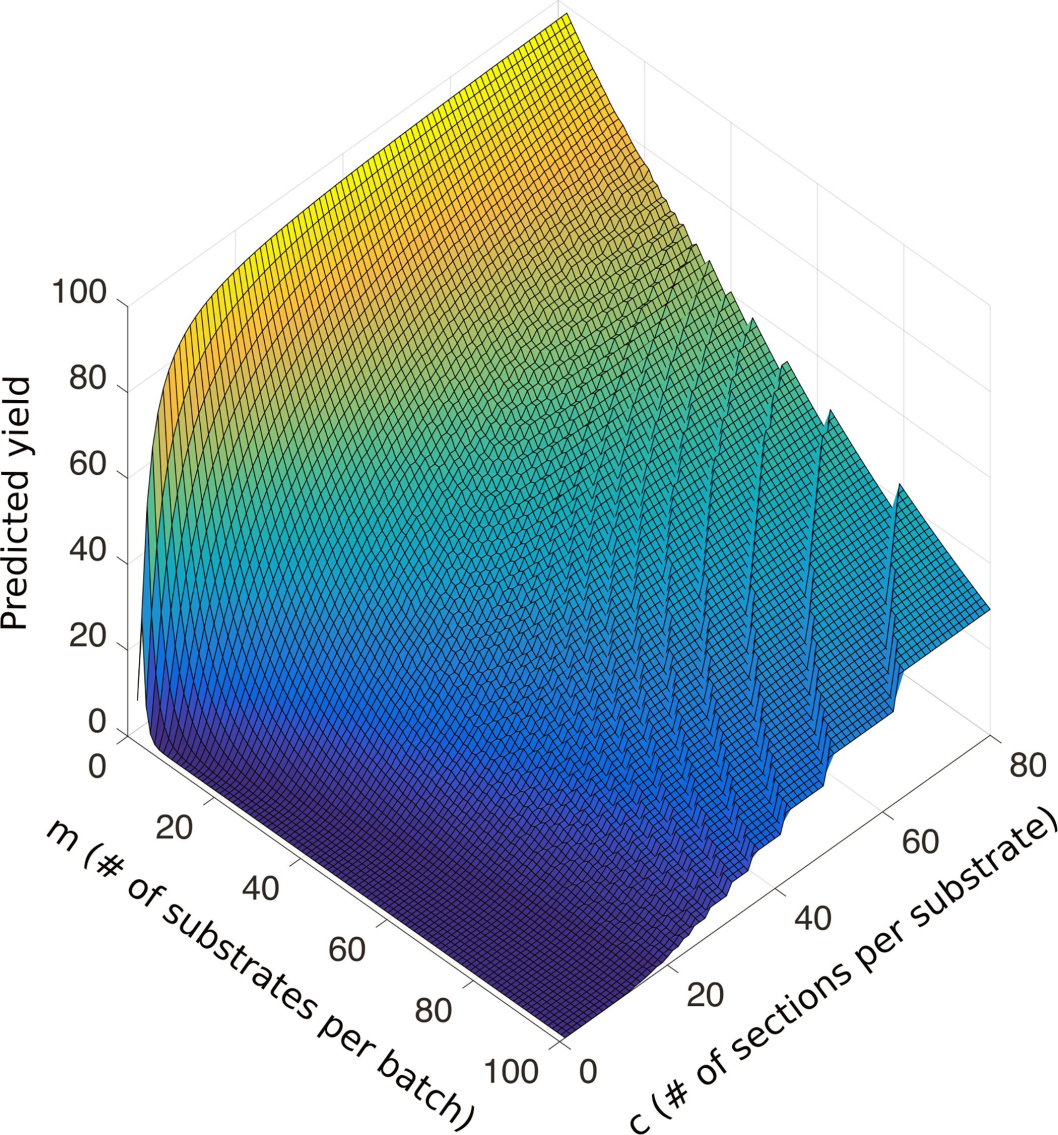
Predicted experiment yield for a batch process as a function of *m*, the number of substrates per batch (1 < *m* < 100) and *c*, the number of sections per substrate (0 < *c* < 80). The upper bounds for *c* and *m* represent practical physical limits while the lower bounds represent minimum physical limits, i.e., for a batch process we mandate at least two substrates per batch with at least one section per substrate.

In developing a mathematical model to predict the likelihood of experiment success (or “yield”) for a large-scale (~1 mm^3^) neuroanatomical study, we implemented a binomial probability-based model with parameters taken from previously published literature. Using Eqs 1–3, we plot in Fig 4 the predicted yield for batch processing 25,000 serial sections, i.e., the likelihood of zero consecutive section loss, as a function of *c*, the number of sections per substrate, and *m*, the number of substrates per batch. We observe the highest yield is attained by minimizing *m* while maximizing *c* (Fig 4, yellow regime). Practically, this corresponds to large EM substrates that can each hold many sections. While this may maximize yield, this optimal solution would be difficult to implement since typical commercial TEMs are designed to hold one grid (~3 mm diameter) with one section on it. Significant modification of a TEM would be required to accept large (>> 3 mm diameter) substrates. Yet, TEM modification is not without precedent, (see references [6] and [13]); thus, the greatest barrier to attaining this theoretical optimum is likely cost. This represents one extreme of our model: a few substrates (*m* ~ 10), each holding many sections (*c* ~ 10^3^–10^4^) maximizes the predicted yield. At the opposite extreme, i.e., having many substrates per batch (*m* ~ 10^4^) with a few sections per substrate (*c* ~ 1), the model predicts the low yield. Intriguingly, this extreme is analogous to traditional serial sectioning, where one section is placed onto its own grid. Thus, our model points towards the theoretical low-yield associated with performing large-scale ssTEM studies via traditional serial sectioning methods (Fig 4, dark blue regime). The step-like behavior of the plot is due to the need for an integer number of batches. Ultimately, this model captures the spectrum on which ssTEM methodologies lie (with current technologies existing only at the extremes), accurately reflects their respective observed yields, and represents a scaffold for further yield optimization.

Furthermore, in modeling ssTEM experiments, we present a mathematical equation that captures the time required to complete a large-scale ssTEM experiment. As shown in Eq 4, in using traditional serial sectioning methods, the total time for data collection scales linearly with the total number of sections to be processed. In order to decrease the total time for data collection, we can either decrease the section load time, imaging time, or pickup time. From prior work, the imaging time can be decreased by implementing a camera array system (e.g., TEMCA [13]). The section pickup time, using traditional serial sectioning methods is slow (~2 min per section, [15]); therefore, in LASSO, by using robotic pickup we decrease the section pickup time, thereby decreasing the total data collection time. We note that *t*_load time, traditional_ ≅ *t*_load time, LASSO_, but expect *t*_load time, LASSO_ to decrease with further multiplexing and automated machine vision algorithms. Additionally, by imaging sections in batches, we are able to decrease the section load time by reducing the number required of TEM pump-down cycles for substrate interchange; this is encapsulated in Eq 5 by increasing *c* and/or *m*. An optimal solution for both yield and throughput may lie in having exactly two substrates, each holding half of the total number of sections. In this case, significant cost would be required to create custom tooling needed for implementation. Adapting technology developed by Hayworth, K., et. al., [16] to ssTEM may be a viable approach to implement this theoretical optimal solution. In total, we find that traditional serial sectioning methods scale poorly with respect to experiment throughput while LASSO, with batch processing and utilization of robotic tooling, can increase experiment throughput.

Lastly, we present two equations to capture the cost associated specifically with the substrates required for traditional serial sectioning methods versus that of batch processing. For traditional serial sectioning, the total cost of substrates scales linearly with the number of sections (see Eq 6). On the other hand, for LASSO, in which sections are processed in batches, we observe in Eq 7 that the cost per section is inversely related to the number of sections per wafer, assuming a wafer-based microfabrication process. Thus, analogous to that of Moore’s law, we predict the cost per section to decrease as the standard wafer size increases, eventually to below the cost of a single grid [28]. In total, these equations demonstrate the feasibility of implementation of LASSO with respect to cost.

In total, our models for yield, throughput, and cost represent a theoretical scaffold for ssTEM experiment optimization. Furthermore as an experimental implementation of LASSO, using Eqs 1–3 and setting *m* and *c* to be 4 and 40, respectively, we predict a ~91% yield, or probability of success, for an ssTEM study with 25,000 sections—an order of magnitude increase in predicted yield using traditional serial sectioning methods. Accordingly, in our experiments, we implement batch processing with *m* and *c* to be 4 and 40 respectively.

### Experiment results

**Fig 5:**
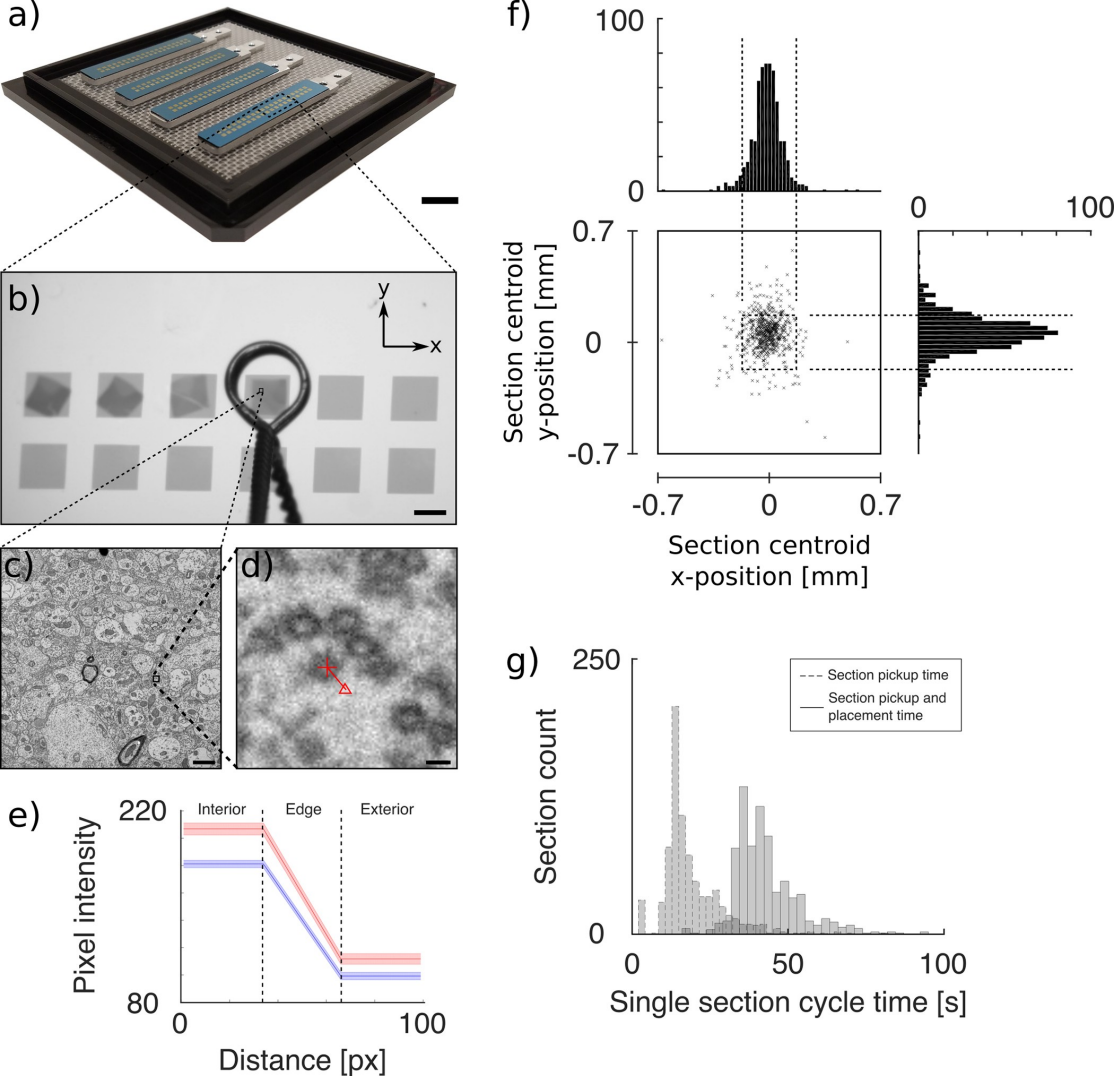
**(a)** Photograph of four microfabricated TEM substrates, each with forty 1.4 mm x 1.4 mm apertures (pitch: p_x_ = p_y_ = 1.9 mm) for TEM imaging. Each aperture contains a 100 nm-thick silicon nitride support films. Scale bar: 10 mm. **(b)** Photograph of a sub-area of a substrate with sections being placed onto the apertures using a loop end-effector. Scale bar: 1 mm. **(c)** Representative transmission electron micrograph of section sub-area on the microfabricated TEM substrate. Scale bar: 1 μm. **(d)** Electron micrograph sub-area depicting labeled vesicle interior (*red cross-hair)*, vesicle exterior *(red triangle)*, and connecting line (*red)* used to measure edge spread function. Scale bar: 10 nm **(e)** Mean edge spread function across manually annotated vesicle edges for images from sections on SiN *(red)* (n = 60) and Luxel *(blue)* (n = 60). We observe no significant difference in the slope of the ESF, indicating comparable sharpness of edges and image quality. **(f)** Using LASSO, scatter plot of section centroid positions with x- and y-centroid position distributions; plot extents correspond to imaging aperture size. Centroids located within the outlined box (*black dashed line*) have their entire area contained within the imaging aperture (587/631 sections, 93%). **(g)** Histogram of single-section pickup and placement time (*solid outline*) and single-section pickup time, only (*dashed outline*).

We fabricated 26 individual silicon/silicon-nitride substrates. A photograph of four substrates (one batch) is shown in Fig 5A. Each substrate contained forty 1.4 mm x 1.4 mm apertures with pitch 1.9 mm in both the x- and y-direction, as shown in Fig 5B. We processed four wafers and obtained an average yield of 81.25% (6.5/8 substrates) for each wafer. We note, while only 23 of the 26 substrates were used in our experiment, the remaining 3 substrates were of usable quality.

We picked up and placed 727 sections across 23 substrates, with a yield of 99.7% (727/729). We find our yield to exceed that of prior work (p < .05), using the Fisher’s exact test, to compare LASSO yield to traditional serial sectioning by Lee, W.A., et. al., (3649/3700 sections) [5]. We note that this comparison is difficult to make due to a variety of uncontrolled parameters (e.g., section size, tissue preparation, sectioning conditions), but in attempting to minimize these differences, we choose to compare to this particular work due to similar section cross-sectional area, section thickness, and the use of mammalian cortical tissue processed for electron microscopy in a similar manner (see [5] for details). The two sections lost were due to substrate aperture failure. To this end, patterning the thin-film with out-of-plane stiffening features (e.g., ribs), may reduce aperture failures. Additionally, alternative aperture materials, such as silicon dioxide or graphene, may further reduce substrate aperture failures. As the field moves towards larger volumes of neural tissue, it is likely that a 1.4 mm x 1.4 mm would be insufficient to encompass the entire section within the imaging aperture. Thus, with the flexibility granted by microfabrication techniques—in particular photolithography—larger apertures can be readily created.

Sparse TEM imaging of sections was conducted to verify image quality. A representative transmission electron micrograph of a section placed on the microfabricated TEM substrate is shown in Fig 5C. Qualitatively, we are able to discern cell membranes and identify individual synaptic vesicles. An image sub-area is shown in Fig 5D, depicting manual annotation of one vesicle using methods described previously. Comparing images obtained on SiN compared to that of Luxel, we observe no visible difference in image quality. Furthermore, the mean slope of the computed edge spread function (ESF) was -2.56 +/-1.07 (n = 60) for Luxel (Fig 5E, blue) and -2.98 +/- 1.83 (n = 60) for SiN (Fig 5E, red), with idealized edges having infinite slope. The contrast for the Luxel film was 0.35 +/- 0.17 (n = 462) and 0.30 +/-0.14 for SiN (n = 636). Our analysis demonstrates that the image quality of the SiN film is comparable to Luxel—a conventionally used TEM grid support film. Thus, LASSO does not adversely affect image quality. Additionally, we observe a slightly larger slope in the SiN ESF, indicating sharper edges in the images. Thus, SiN-based substrates may be a suitable substrate for automated segmentation algorithms [29, 30]. While the Luxel support film does exhibit higher contrast, commonly used contrast enhancing methods (e.g., histogram equalization) could be applied to improve the contrast for SiN substrates.

Applying the criteria previously outlined, we analyzed the section placement accuracy and repeatability for 631/729 sections and report accuracy and repeatability of -20 ± 110 μm (x-axis) and 60 ± 150 μm (y-axis), as shown in Fig 5F. We find that 587/631 (93%) sections lie completely within imaging aperture while the remaining 631–587 = 44 sections are partially occluded from the imaging aperture, i.e., roughly 7% (44/631) sections lie outside of the imaging aperture. While traditional definitions of section loss would consider these 44 sections to be “lost,” we assert that this failure modality is easily remedied via fabrication of substrates with larger imaging apertures. From sections analyzed, given the same accuracy and repeatability, all 631 sections would lie within the 3 mm x 3 mm imaging aperture. We note the 96 sections excluded (due to their breaking into multiple pieces) from our accuracy and repeatability analysis (727–631 = 96 sections) may have fragmented due to a variety of causes, but we do not see this as a fundamental barrier for LASSO due to our ability to successfully pick up and place these sections onto TEM substrates as well as developments in segmentation algorithms to recombine fragmented sections *in silico* [11]. Further study may investigate potential causes for section fragmentation to prevent partial section all together. Potential causes may include loop geometry, hysteresis of the water with respect to the loop, embedding plastic material properties, embedment protocol, or experiment section dry-down conditions.

We report an average cycle time of 43.5 s ± 11.7 s (Fig 5G, solid outline) and an average section pickup time of 18.9 s ± 9.7 s (Fig 5G, dashed outline). As a first order approximation, we observe a section dry-down time of 24.6 s, calculated as the average cycle time minus the average section pickup time. This is likely an over estimation since this value includes the time required for travel to and from the waterboat. In comparing our average section pickup time that of from prior work (~2 min/section [15]), we find that our methodology has decreased the section pickup time by approximately a factor of five while removing the need for a human user with expert dexterity to pick up serial sections. Additionally, as demonstrated in electrophysiology [31], further automation of section pickup and multiplexing could lower the cycle time even further; thus increasing experiment throughput. Use of machine vision algorithms may accelerate the identification of section centroids while implementation of an automated section transport mechanism may obviate the need for a manually actuated air needle. Automated transport of sections away from the knife-edge prior to section pickup may be accomplished via an automated pneumatic system, standing surface acoustic waves [32] or quadrupolar capillary interactions [33].

LASSO, as previously described, is the composition of several independent technologies that together create flexible, scalable, and accessible platform for large-scale ssTEM. Yet, each technology on its own merits its own discussion. As a part of LASSO, we introduce batch processing, an industrial engineering ideology commonplace in large-scale manufacturing settings. While we demonstrate and characterize a batch processing scheme that utilizes robotic tools and microfabricated substrates, these specifics components are not required to implement batch processing. Moreover, as shown from our modeling results, it is arrangement of serial sections in quantized groupings in a non-consecutive order that enables higher yield. This could be done whether using tape-based substrates [16], silicon wafer substrates [17], or glass slides [34]. By distributing the risk of losing consecutive sections by physically placing them on separate substrates, we maximize the overall experiment yield.

Furthermore, in our implementation of LASSO, we used silicon/silicon-nitride substrates. In their current state, these substrates could be amenable to automated multi-parameter analysis by combining ssTEM with other analysis methods such as electrophysiology [31], array tomography [34, 35], and genetic analysis [36]. Alternatively, these substrates could used with other imaging modalities, such as multi-beam scanning electron microscopy (SEM) [37], scanning transmission electron microscopy (STEM) [38], or x-ray microscopy [39], to provide imaging at multiple resolutions. In this manner, coarse rapid imaging could be combined with high-resolution TEM for morphological alignment. Moreover, further work could explore augmentation of our substrates. A promising direction may be the incorporation of micro-channels within the substrate [40] or as a separate PDMS device [41] to enable *in situ* staining. Additionally, the substrates could be designed for cell-culture to enable on-chip live-cell imaging/electrophysiology followed by live-cell EM imaging [42, 43]. While each of these are potential future directions, LASSO does not depend on the utilization of microfabricated substrates. In our implementation, the substrates could be readily replaced with tape-based substrates, traditional grids, silicon wafers, or other substrates of choice.

While we demonstrate the ability to manipulate ultrathin sections, LASSO is likely compatible with a variety of section thicknesses. Therefore, semi-thin sections (~100 nm) for light microscopy or EM-based tilt tomography as well as thick sections (~10 μm) for focused ion beam scanning electron microscopy (FIBSEM) could be collected and placed onto an appropriate substrate using LASSO.

While this study was limited to 729 sections, we do not expect fundamental barriers for scaling this technology to 10^3^ to 10^5^ sections, given appropriately apportioned batches. While other technologies may achieve high-yield and high-throughput serial sectioning (see [16, 17]), the accessibility of LASSO, due our use of commercially available cameras, linear actuators, open-source, python-based control software, and standard microfabrication techniques, is a favorable alternative for large-scale serial sectioning.

## Conclusion

LASSO represents a flexible, scalable, and accessible technology platform to enable the next generation of large-scale neuroanatomical ssTEM studies. From our modeling, we find that LASSO exceeds the yield, the throughput, and potentially, the cost of traditional serial sectioning methods. Moreover, implementing a batch size of four substrates, with each substrate holding forty sections, we predict an order of magnitude increase in yield. Using this prediction, we microfabricated custom substrates with corresponding size and implemented LASSO to quantify the yield and throughput of the methodology. We find our yield 727/729 (99.7%) exceeds that of prior work (two-sided Fisher test, p = .05); sections were placed accurately and repeatably (x-direction: -20 ± 110 μm (1 s.d.), y-direction: 60 ± 150 μm (1 s.d.)) within the imaging aperture. Sparse TEM imaging of sections showed no significant distortion, high-frequency information loss, or substrate-derived artifacts resulting from serial sectioning via LASSO. Regarding throughput, we find our methodology decreased the section pickup time by a factor of five while removing the need for a human user with expert dexterity to pickup serial sections. (Mean cycle time: 43.5 s ± 11.7 s; mean section pickup time: 18.9 s ± 9.7 s). This technology demonstrates a powerful tool for automating serial sectioning—a significant bottleneck for ssTEM neuroanatomical studies. Thus, we envisage LASSO will enable ssTEM physiological and neuroanatomical studies that investigate neural tissue volumes of size previously not possible, thereby bringing significant insight into the field of neuroscience.

## Supporting information

S1 Protocol Fabrication protocol for TEM substrates. (PDF)

S1 Movie Example of section pickup and placement process. (AVI)
