# Supplementary material for: Large-scale neuroanatomy using LASSO: Loop-based Automated Serial Sectioning Operation
Source: PLoS One. 2018 Oct 23;13(10):e0206172. doi: 10.1371/journal.pone.0206172 (PMC6198950; doi:10.1371/journal.pone.0206172)
Supplement: S1 Protocol — (PDF) [file pone.0206172.s001.pdf]

STEP #	PROCESS DESCRIPTION	CHECK-OFF
0010	**OBTAIN SILICON WAFER WITH SiN**<br><br>POLISH: SSP<br>DIAMETER: 4 INCH<br>DOPANT: B<br>TYPE: P<br>ORIENTATION: <100><br>THICKNESS: 500 μm<br>GRADE: PRIME<br><br>SiN SPECIFICATIONS:<br><br>THICKNESS: 100 nm<br>TYPE: LOW-STRESS CVD<br><br>VENDOR: UNIVERSITYWAFER.COM	
0020	**DEPOSIT HMDS LAYER**<br><br>EQUIPMENT: KARL SUSS RC8 SPINNER (MARCUS INORGANIC CLEANROOM)<br><br>RECIPE: STEP 1: 3000 rpm<br>1000 rpm/s<br>10 s<br><br>NOTES FOR KARL-SUSS RC8 SPINNER:<br><br>• PRESS ‘ENTER’ TO CLEAR ERRORS<br>• PRESS ‘ST/STOP’ TO CLEAR ERRORS UNTIL YOU SEE ‘0000’<br>• ‘DEVICES’ SHOULD ALL BE ‘READY’<br>• RIGHT-CLICK TO RESET IF THERE ARE STILL ERRORS<br>• IF YOU NEED TO, POWER CYCLE THE MACHINE<br>• SELECT THE OPEN FOLDER ICON TO ADD A RECIPE<br>• SELECT THE NEW PAGE ICON TO EDIT A RECIPE	

	• TO RUN YOUR RECIPE, USE THE DROP-DOWN MENU TO SELECT YOUR RECIPE, THEN SELECT RUN<br>• BE SURE TO RUN THE AUTO-CLEAN RECIPE WITH THE CLEANROOM WAFER<br>• FOR THE HOT-PLATE, DO NOT CHANGE 'CALIBRATION' OR 'PID' LOOPS VALUES	
0030	**DEPOSIT PHOTORESIST**<br><br>PHOTORESIST: SPR-220<br><br>EQUIPMENT: KARL SUSS RC8 SPINNER (MARCUS INORGANIC CLEANROOM)<br><br>RECIPE: STEP 1: 500 rpm<br>100 rpm/s<br>10 s<br><br>STEP 2: 3000 rpm<br>1000 rpm/s<br>40 s<br><br>STEP 3: 0 rpm<br>500 rpm/s<br>0 s	
0040	**BAKE PHOTORESIST**<br><br>EQUIPMENT: KARL SUSS RC8 SPINNER (MARCUS INORGANIC CLEANROOM)<br><br>RECIPE: TEMPERATURE: 115 °C<br>TIME: 60 seconds	
0050	**ALIGN MASK / EXPOSE PHOTORESIST**<br><br>EQUIPMENT: KARL SUSS TSA MA-6 (MARCUS INORGANIC CLEANROOM)	

	PARAMETERS:<br><br>CONTACT TYPE: HARD CONTACT<br>ALIGNMENT GAP: 30 µm<br><br>NOTES FOR KARL-SUSS TSA MA-6:<br><br>• BE SURE TO CHECK LAMP INTENSITY WITH UV SENSOR, IN BLACK CASE UNDERNEATH MACHINE<br>• BE SURE 365 nm WAVELENGTH IS SELECTED<br>• CH 1 IS 365 nm<br>• IF 'CHANGE MASK' SCREEN IS ON, PRESS' CHANGE MASK' TO GO BACK TO MAIN SCREEN<br>• PLACE SENSOR ON CHUCK AND PRESS 'LAMP TEST' TO COMMENCE UV INTENSITY TEST<br>• FOR SPR-220, APPROXIMATELY 500 mJ/cm^2 IS REQUIRED<br><br>• USE 'EDIT PARAMETER' BUTTON TO ADJUST EXPOSURE TIME AND THE ALIGNMENT GAP<br>• HARD CONTACT IS OK<br>• ONCE MASK IS ALIGNED, PRESS 'ALIGN CONT/EXP' TO BRING WAFER INTO CONTACT WITH MASK. IF ALIGNMENT IS NOT GOOD, PRESS 'ALIGN CONT/EXP' TO RELEASE MASK AND WAFER PROCEED WITH ALIGNMENT<br>• PRESS EXPOSE TO BEGIN EXPOSURE.<br><br>• BE SURE TO REMOVE THE MASK, RETURN SUBSTRATE KNOBS TO X10 AND Y10, AND ROTATE CHUCK HOLDER SO THAT THE WHITE LINES MATCH.	
0060	**DEVELOP PHOTORESIST**<br><br>DEVELOPER: MF-319 for SPR-220 photoresist<br><br>DEVELOP FOR 3 MINUTES, THEN PLACE WAFER IN WATER BATH FOR AT LEAST 3 MINUTES. AFTERWARDS, THOROUGHLY DRY THE WAFER.	

0070

ETCH SILICON NITRIDE LAYER THRU TO SILICON

EQUIPMENT: OXFORD END-POINT RIE (MARCUS INORGANIC CLEANROOM)

NOTES FOR OXFORD END-POINT RIE:

- VISION RIE IS FINICKY. IT MIGHT BE FASTER, BUT IT IS INCONSISTENT. BEST TO USE OXFORD.
- SELECT THE ‘SYSTEM’ BUTTON
- PRESS ‘STOP’ TO BEGIN VENTING PROCESS
    - SELECT ‘VENT’ TO VENT CHAMBER
- WHEN TIME IS <100s, SWITCH ‘CHAMBER DOWN’ TO ‘CHAMBER UP’
    - THEN PRESS BOTH GREEN BUTTONS SIMULTANEOUSLY TO OPEN CHAMBER
    - YOU WILL ALSO HEAR WHEN IT IS OK TO OPEN THE CHAMBER
- MAKE SURE O-RING IS SEATED EVENLY
- THERE ARE 2 PLATTENS: Al & Graphite
    - Al if etch depth is < 2 µm
    - Graphite is etch depth is > 2 µm
- SWITCH TO ‘CHAMBER DOWN’ AND PRESS BOTH GREEN BUTTONS SIMULTANEOUSLY TO LOWER LID
    - MAKE SURE LID IS ALIGNED CORRECTLY. MAY NEED TO ADJUST MANUALLY
- PRESS ‘STOP’, THEN ‘EVACUATE’
    - BE SURE TO READ PROMPT CLOSELY. IT WILL TELL YOU TO PRESS ‘CANCEL’ IF YOU ARE EVAUCATING AN EMPTY CHAMBER.
- TO SELECT YOUR PROCESS, GO SELECT THE PROCESS BUTTON
    - RECIPES -> LOAD -> OK

- **STANDARD OXIDE ETCH: (OK for etching 100 nm of SiN)**
    - **PRESSURE: 50 mTorr**
    - **O_2: 4**
    - **CHF_3: 35**
    - **RF power: 250**
    - **Step time: 3 min**

- AFTER DONE, SELECT CHAMBER CLEAN
    - MAKE SURE Al PLATTEN IS IN CHAMBER

<table>
<tr><td>0080</td><td>ETCH (DRIE) SILICON

EQUIPMENT: STS HRM ICP (MARCUS INORGANIC CLEANROOM)

RUN RECIPE ‘HC_GENM’ FOR 350 CYCLES. THIS SHOULD ETCH 400 µm OF Si.

NOTES FOR STS HRM ICP:
‘VENT’ LOAD LOCK TO LOAD SAMPLE; LID WILL OPEN ONCE BY ITSELF
PLACE SAMPLE ON SHUTTLE, CLOSE LID, AND PRESS ‘PUMP & MAP’
SELECT WAFER POSITION (1 OR 2) AND PRESS ‘LOAD’ TO LOAD SAMPLE INTO ETCHING CHAMBER
GO TO ‘RECEIPE’
OPEN AND EDIT THE RECIPE YOU WANT TO RUN.
IT IS OK TO OVERWRITE RECIPES
USE ‘HC_GENM’ RECIPE
WE WILL ONLY EDIT THE NUMBER OF CYCLES
350 CYCLES ≈ 400 µm
SAVE RECIPE AND CLOSE RECIPE EDITING WINDOW
PRESS ‘SELECT’ (NOT ‘PROCESS’)
SELECT DESIRED RECIPE, LET IT LOAD. ONCE LOADED, PRESS ‘PROCESS’ TO BEGIN ETCH.</td><td></td></tr>
<tr><td>0090</td><td>SUBMIT WAFERS TO DICING SERVICE WITH CHARLIE SUH. <suh@gatech.edu>

PLACE WAFERS IN STAFF ROOM CABINET LABELED ‘DICING SAMPLES’

BE SURE TO SUBMIT DICING INSTRUCTIONS TO ENSURE CORRECT DICING</td><td></td></tr>
<tr><td>0100</td><td>PEEL INDIVIDUAL DICED SUBSTRATES FROM DICING TAPE

SUBSTRATES ARE VERY FRAGILE; USE CARE WHEN PEELING SUBSTRATES OFF OF DICING TAPE.

USING ACETONE TO REMOVE ADHESIVE CAN HELP BUT CAUSES ADHESIVE LAYER TO COME OFF OF THE TAPE BACKING. CAN BE MESSY.</td><td></td></tr>
</table>

0110	**RINSE SUBSTRATES OFF WITH ACETONE THEN ISOPROPYL ALCOHOL AND THEN DI WATER**<br><br>BE SURE TO REMOVE ALL RESIDUAL PHOTORESIST PRIOR TO KOH ETCH.	
0120	**LET SUBSTRATES AIR DRY THOROUGHLY**<br><br>SUBSTRATES CAN BE PLACED ON HOTPLATE TO ACCELERATE DRYING.	
0130	**KOH ETCH SILICON THRU TO SILICON NITRIDE AT 85˚C FOR 1 HOUR OR UNTIL APERTURES ARE CLEAR.**<br><br>EQUIPMENT: KOH 45% AQUEOUS SOLUTION<br>TEFLON ETCHING FIXTURE<br><br>DO NOT DILUTE KOH; IT SHOULD BE AT CORRECT CONCENTRATION.<br><br>PLACE SUBSTRATES IN TEFLON ETCHING FIXTURE. EXPOSED SILICON SHOULD BE FACING UP TO ALLOW HYDROGEN BUBBLE TO FREELY PERCOLATE. FIXTURE HOLDS UP TO 9 SUBSTRATES<br><br>PLACE FIXTURE WITH SUBSTRATES IN SHALLOW BEAKER AND FILL WITH KOH TO FULLY COVER FIXTURE<br><br>DO NOT OVER ETCH SUBSTRATES. SILICON FRAME MAY TO DETERIORATE	
